# Frequent Use of Baby Food Pouches in Infants and Young Children and Associations with Energy Intake and BMI: An Observational Study

**DOI:** 10.3390/nu16183165

**Published:** 2024-09-19

**Authors:** Neve H. McLean, Bailey R. Bruckner, Anne-Louise M. Heath, Jillian J. Haszard, Lisa Daniels, Cathryn A. Conlon, Pamela R. von Hurst, Kathryn L. Beck, LA Te Morenga, Ridvan Firestone, Jenny McArthur, Rebecca Paul, Alice M. Cox, Emily A. Jones, Ioanna Katiforis, Kimberley J. Brown, Maria Casale, Rosario M. Jupiterwala, Madeleine M. Rowan, Andrea Wei, Louise J. Fangupo, Megan Healey, Veisinia Pulu, Tia Neha, Rachael W. Taylor

**Affiliations:** 1 Department of Human Nutrition, University of Otago, Dunedin 9016, New Zealand; neve.mclean@otago.ac.nz (N.H.M.); bailey.bruckner@otago.ac.nz (B.R.B.); jenrose19@hotmail.com (J.M.); ioanna.katforis@otago.ac.nz (I.K.); maddie.rowan@otago.ac.nz (M.M.R.); 2 Biostatistics Centre, University of Otago, Dunedin 9016, New Zealand; jill.haszard@gmail.com; 3 Department of Medicine, University of Otago, Dunedin 9016, New Zealand; lisa.daniels@otago.ac.nz (L.D.); alice.cox@otago.ac.nz (A.M.C.); lou.fangupo@otago.ac.nz (L.J.F.); rachael.taylor@otago.ac.nz (R.W.T.); 4 School of Sport, Exercise and Nutrition, Massey University, Auckland 0632, New Zealand; c.conlon@massey.ac.nz (C.A.C.); p.r.vonhurst@massey.ac.nz (P.R.v.H.); k.l.beck@massey.ac.nz (K.L.B.); r.paul@massey.ac.nz (R.P.); e.jones@massey.ac.nz (E.A.J.); dietitian.kimberly@outlook.com (K.J.B.); m.casale@massey.ac.nz (M.C.); r.p.monzales@massey.ac.nz (R.M.J.); a.wei@massey.ac.nz (A.W.); 5 Research Centre for Hauora and Health, Massey University, Wellington 6140, New Zealand; l.t.morenga@massey.ac.nz (L.T.M.); r.t.firestone@massey.ac.nz (R.F.); m.healey@massey.ac.nz (M.H.); v.pulu@massey.ac.nz (V.P.); 6 School of Psychology, Victoria University of Wellington, Wellington 6140, New Zealand; tia.neha@vuw.ac.nz

**Keywords:** infant, baby food pouch, food pouch, energy intake, body mass index

## Abstract

Objective: Most wet commercial infant foods are now sold in squeezable ‘pouches’. While multiple expert groups have expressed concern about their use, it is not known how commonly they are consumed and whether they impact energy intake or body mass index (BMI). The objectives were to describe pouch use, and determine associations with energy intake and BMI, in infants and young children. Methods: In this observational cross-sectional study of 933 young New Zealand children (6.0 months–3.9 years), pouch use was assessed by a questionnaire (‘frequent’ use was consuming food from a baby food pouch ≥5 times/week in the past month), usual energy intake using two 24-h recalls, and BMI z-score calculated using World Health Organization standards. Results: The sample broadly represented the wider population (27.1% high socioeconomic deprivation, 22.5% Māori). Frequent pouch use declined with age (infants 27%, toddlers 16%, preschoolers 8%). Few children were both frequent pouch users and regularly used the nozzle (infants 5%, toddlers 13%, preschoolers 8%). Preschoolers who were frequent pouch users consumed significantly less energy than non-users (−580 kJ [−1094, −67]), but infants (115 [−35, 265]) and toddlers (−206 [−789, 378]) did not appear to have a different energy intake than non-users. There were no statistically significant differences in the BMI z-score by pouch use. Conclusions: These results do not support the strong concerns expressed about their use, particularly given the lack of evidence for higher energy intake or BMI.

## 1. Introduction

Baby food pouches are becoming an increasingly popular way to assist the transition from a solely human milk or infant formula diet to solid foods, both in New Zealand and elsewhere [1]. Worldwide, their market share is growing [2], so that they now represent more than half of all infant commercial foods in many countries [1,3]. Food pouches are flexible food containers with a nozzle and a re-screwable cap that young children could use to feed themselves [4]. The foods in pouches need to be puréed so that they can be squeezed through the nozzle. This texture is consistent with recommendations for introducing solids to infants [5], and most of these pouches are aimed at the infant market, but some are marketed to older age groups. Anecdotal reports suggest that it is also likely that baby food pouches are being used well beyond 12 months of age.

Surprisingly, how baby food pouches are being used by infants and young children is currently unknown. While the contents are largely similar to foods in other commercial wet food containers such as jars and cans [1], it is the potential for the food to be sucked directly through the pouch nozzle that makes them different to traditional commercial infant foods. This design accommodates feeding away from home, facilitates infant self-feeding [4], and the ‘convenient, mess-free’ packaging may be seen by parents as a practical approach both to complementary feeding [2,6] and to feeding older age groups who are still learning to use eating utensils without mess. However, these features have also prompted concerns that pouches may change the way solid foods are consumed, with some expert groups cautioning against their use, believing that the regular sucking of sweet, semi-liquid purées from pouches could have detrimental effects on oral motor development, dental health, and obesity risk [4,7,8,9]. Despite these concerns, little evidence exists showing how food pouches are actually being used [10] and whether they impact energy intake or body weight in infants and young children [11,12]. Lundkvist et al. [11] reported that pouch consumption was not related to the body mass index (BMI) z-score at 18 months of age, but they only assessed fruit pouches, and did not report on energy intake. Cox et al. [12] demonstrated that frequent pouch use was not related to energy intake or BMI z-score in infants but had no data in older children. 

The aims of this study were to (1) describe the use of food pouches in infants, toddlers, and young preschoolers and (2) determine whether frequent use was associated with energy intake and body mass index (BMI) in young children aged 6 months to 3.9 years.

## 2. Materials and Methods

Data for these analyses were obtained from the First Foods New Zealand (FFNZ) and Young Foods New Zealand (YFNZ) observational, cross-sectional studies of feeding practices, nutrition and health in children aged 6 months to 3.9 years. Ethical approval for FFNZ was obtained from the Health and Disability Ethics Committees New Zealand (19/STH/151) and for YFNZ from the University of Otago Human Research Ethics Committee (H20/040). In both studies, all adult participants provided written informed consent. FFNZ was a more comprehensive study than YFNZ, and a protocol paper is available [13]. FFNZ was registered (prospectively) with the Australian New Zealand Clinical Trials Registry (ACTRN12620000459921) given the more complex nature of data collection involved. The sample size of 625 in FFNZ (participants 7.0–9.9 months of age) allowed us to estimate the prevalence of frequent baby food pouch use to a 95% precision level of at most ±4% [14]. In YFNZ (participants 6.0 months–3.9 years of age), we aimed to recruit 300 participants which allowed a 95% precision interval for the estimation of the prevalence of pouch use to, at most, ±6%.

Participants were child and main carer pairs who lived in Dunedin or Auckland (main urban centres in the South and North Islands of New Zealand) or Wellington (capital city of New Zealand). Children had to be 6 months to 3.9 years of age and not have recently participated in an intervention that might have influenced their eating. Adult participants were 16+ years of age and could communicate in English. Participants were recruited through advertisement or word of mouth (July 2020–February 2022). Care was taken to target all children rather than care givers focusing on specific infant feeding practices to minimise bias.

Participants attended two appointments with a trained researcher. The first appointment included a main questionnaire (demographics, pouch use), a 24-h recall, and anthropometric measurements, and the second appointment included a second 24-h recall. Demographic information collected at the first appointment included adult participant age, maternal parity, and self-reported maternal height and weight; infant sex and ethnicity (prioritised ethnicity using Census Level 2 reporting in which ethnicity is classified in the following order: Māori, Pacific, Asian, Other, European [15]); and area-level socioeconomic deprivation (New Zealand Index of Deprivation [NZDep] using the participant’s home address as a proxy indicator of socioeconomic status, with 1 indicating the lowest level of socioeconomic deprivation, and 10 the highest [16]).

Pouch use was measured using questions investigating the frequency and method of pouch consumption. These questions had been designed by the research team in the absence of published studies on food pouch consumption patterns. Pouch use questions included whether the child had ever eaten food from a commercial baby food pouch, the frequency of use, the extent to which the child sucked the food directly from the pouch or was spoon-fed the contents, how often home-filled (i.e., reusable) pouches were used, where pouches were typically consumed, and the extent to which the child was supervised by an adult when eating from a pouch. We asked caregivers to answer these pouch questions referring to the child’s current age. Caregivers were asked why they used food pouches, with multiple options to choose from as well as a free text space to write any other reasons. Caregivers of the infant cohort who took part in the FFNZ study (n = 625) were also asked about pouch use retrospectively for when the infant first started eating solids, and retrospectively for when the infant was around 6 months of age, as well as currently at the time of assessment.

Energy intake was assessed using two interviewer-administered multiple pass 24-h dietary recalls collected by trained researchers, and measured human milk intake, as described below. Where possible, diet recalls were administered on different days of the week to capture the daily variation in intake. Caregivers were asked to take photographs of their infant’s food and beverages (including infant and follow-up formulas) on the day before the recall, to act as a memory prompt. The 24-h recall three-pass method was used where the first pass captured a ‘quick-list’, in which the caregiver listed each food or liquid (and breastfeed) that had been offered to the infant the previous day. The second pass captured additional detail, including a description of each item (including the brand and preparation method used), and the amount offered and consumed using visual aids such as dishes and utensils to estimate amounts if needed. The third pass consisted of a review process and probing for forgotten items. Quality checks were undertaken at regular intervals during the study to ensure adherence to data collection protocols.

For infants who were breastfed, the energy intake from breast milk was determined by the direct measurement of breast milk volume or the use of predictive equations. Accurate data on breast milk intake were determined in a subsample of FFNZ participants (n = 157) using the deuterium oxide dose-to-mother stable isotope technique [17,18,19]. This method measures the disappearance of deuterium in the saliva (representing total body water) of the mother and appearance of deuterium in the infant over a 14-day period following administration of a dose of deuterium oxide to the mother. The measures obtained provide a measure of breast milk intake at the individual level, accurate to 3% of estimated intakes of 787 mL/day [20]. For the breastfed infants under 12 months of age who were not in the subsample, the estimated daily breast milk intake in grams per day was calculated using a predictive equation developed using the dose-to-mother dataset—the Human Milk Intake Level Calculation (HuMILC) tool 1 [21]. For children 12 months of age and over, the breast milk intake was estimated as 91.4 g per feed if aged between 12 and 18 months or 60.9 g per feed if older than 18 months [22]. Breast milk intakes were then converted to energy intake using a nutrient density of 264 kJ/100 g (Subathira Sivakumaran, personal communication).

Dietary data were analysed using the nutrient analysis software programme FoodWorks (version 10, Xyris Software, Brisbane, Australia) and nutrient data from the New Zealand Food Composition database FOODfiles 2018 Version 01 [23]. Data on commercial infant foods (which are not available in FOODfiles) were obtained from an extensive database of nutrient profiles, previously established by the researchers [1] and updated for new foods using the same processes.

For toddlers (12.0–23.9 months of age) and preschoolers (24.0 to 47.9 months of age), the ‘usual’ energy intake from all foods and drinks was calculated from the two 24-h recalls using the Multiple Source Method [24,25] which statistically adjusts for the day-to-day variation in nutrient intake. For infants (6.0–11.9 months of age), usual energy intake from breast milk and infant formula were determined separately as described below, and these values were then added to the usual energy from complementary foods which was calculated from the two 24-h recalls using the Multiple Source Method [24,25]. The deuterium oxide dose-to-mother method described above provides a measure of breast milk intake over 14 days, so daily breast milk intake calculated from this value was considered to reflect the infant’s current usual breast milk intake. The infant’s average daily energy from infant formula was calculated using volume data from the 24-h recalls and specific infant formula composition data extracted from product nutrition information panels. The toddler and preschooler intakes of breast milk were determined as described above, and formula intake was determined in the same way as for infants.

Child weight, and length or height, were measured in duplicate by a trained researcher following standard protocols which varied depending on the child’s age [26]. In general, children under 24 months of age had their weight measured on infant scales (Seca, Models 224 and 354, Hamburg, Germany) and their length measured on a baby measuring mat (Seca, Model 210, Hamburg, Germany). Children 24 months or older had their weight measured on adult scales (Seca, Model 354, Hamburg, Germany) and height measured standing on a portable stadiometer (Model WMHM200P, Wedderburn, NZ). BMI-for-age z-scores were determined separately in boys and girls using the World Health Organization Child Growth Standards [27].

Data were analysed using Stata version 18.0 statistical software [28]. Infants, toddlers, and preschool-aged children were classified as *frequent pouch users* if they were given a commercial food pouch at least 5 times/week in the past month; *less frequent pouch users* if they had been given a pouch in the past month but less than 5 times/week; and *non-users* if they had not used a pouch in the past month (or ever). The prevalence of ever having had a pouch, and frequent pouch use, in the NZ population was estimated with a logit-transformed 95% confidence interval (CI) by weighting these results using inverse probability weighting for ethnicity and NZDep using data from a national cohort of infants from 2019 [29].

The mean differences and 95% CI were estimated for energy intake and BMI z-score by frequency of pouch use using linear regression models, both unadjusted and adjusted for age and area-level socioeconomic deprivation. As pouches are a complementary food and there can be demographic and energy intake differences in infants who consume breast milk and those who consume infant formula, differences in energy intakes from complementary foods only were also assessed for the infant subgroup. Residuals were plotted and visually assessed for homoskedasticity and normality. *p*-values < 0.05 were considered statistically significant.

## 3. Results

**Figure 1:**
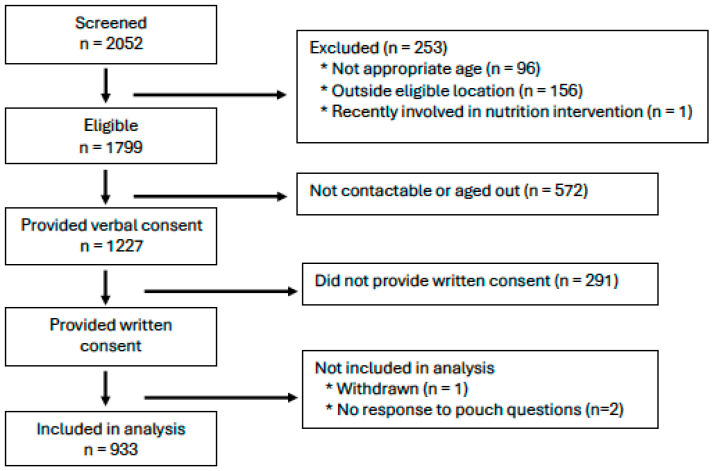
Consort flow chart of participants in FFNZ and YFNZ combined.

The participant characteristics of the final sample (Figure 1) are shown in Table 1. The sample was about half female (47.7%) and ethnically diverse, with 22.5% identifying as Māori (indigenous people of New Zealand), 10.3% as Pacific, 13.7% as Asian, and 51.1% as European. These figures compare well with population data for this age group in New Zealand of 16% Māori, 11% Pacific, 25% Asian, and 63% European [29]. There was also considerable diversity in socioeconomic status, with 28.6% of children living in areas of low deprivation and 27.1% in areas of high deprivation (national figures for New Zealand are 31% and 29%, respectively [29]). Large proportions of infants (66.1%) and toddlers (40.6%), and a much lower proportion of preschoolers (6.6%), were currently breastfed. The mean (SD) BMI-for-age z-score across the group was 0.41 (1.04). In terms of employment, 33.7%, 55.7%, and 51.1% of caregivers (almost all of whom were mothers) were in paid employment (part-time or full-time).

Most infants (78.5%) had eaten from a baby food pouch at least once by the time they participated in the study (mean age 8.4 months). This was higher for toddlers (87.7%; mean age 18.3 months) and preschoolers (84.1%; 36.8 months). The prevalence estimates were only slightly different when weighted to represent the New Zealand population more closely: infants 77.5% (95% CI 73.9, 80.7), toddlers 91.0% (83.4, 95.4), and preschoolers 84.6% (77.7, 89.8). Almost half (47.8%) of the FFNZ infant cohort were reported to have used a pouch at least once at 6 months of age, and just over a quarter (26.7%) to have used one at least once when solids were first introduced (mean [SD] age 5.2 [0.7] months. Most of the pouches used were commercial baby food pouches, but 8.7% of infants, 5.7% of toddlers, and 3.3% of preschoolers were frequently using a reusable, or home-filled, pouch at the child’s current age.

Just over one quarter (27.4%) of infants, 16% of toddlers, and 8% of preschoolers were classified as current frequent pouch users (Table 2). Weighted prevalence estimates were 25.8% (95% CI 22.5, 29.5) for infants, 12.6% (7.1, 21.3) for toddlers, and 8.4% (4.8, 14.4%) for preschoolers. Fewer than one in five infants (17.3%) consumed the pouch contents directly through the nozzle (always or mostly), whereas this was considerably more common in the older age groups (76.3% toddlers, 79.7% preschoolers). However, when considering both frequency and the method of intake, only 20 infants who were frequent pouch users ‘always’ or ‘mostly’ sucked the baby food directly from the pouch; equating to 3% (20/645) of infants overall. While the comparable proportions were considerably higher in toddlers and preschoolers, these percentages should be viewed with caution considering the low absolute numbers of children consuming food from pouches via using the nozzle on a frequent basis. At 6 months of age (collected for the FFNZ infant cohort), just 3% (19/625) always or mostly consumed food through the nozzle, and just 2% (14/625) were also frequent users.

Some differences in usual location of pouch consumption were apparent by age; most infants consumed these pouches while sitting in a seat, with relatively few doing so while ‘on-the-go’ (11.8%), compared with more than one-third of toddlers and preschoolers. Considerably more caregivers ‘always’ supervised their infant when they were consuming a pouch (87.1%) than toddlers (34.3%) or preschoolers (14.9%) (Table 2).

Table 3 compares energy intake and BMI z-score according to the frequency of baby food pouch use. Overall, pouches contributed a median of 287 kJ to the total energy intake in infants and 343 kJ in toddlers. Comparable data could not be calculated for preschoolers because of the low sample size. In infants, the usual daily energy intake from the whole diet did not differ significantly across frequency categories, although less frequent pouch users (fewer than 5 times per week in the past month) consumed significantly more energy (137 kJ; 95% CI 10, 264) from complementary foods than non-users. Broadly similar, but not statistically significant, figures were observed for those infants classified as frequent users (120 kJ; −23, 263). However, there were no significant differences in the BMI z-score between less frequent (mean; 95% CI: 0.07; −0.12, 0.27) or frequent (0.17; −0.05, 0.39), pouch users and non-users. In toddlers, both less frequent and frequent pouch users tended to consume less energy and have lower BMI z-scores overall than non-users, but the differences were not significant, and the confidence intervals were wide because of the relatively small numbers in this age group. Lastly, preschoolers who were frequent consumers of pouches consumed significantly less energy each day than non-users (−515 kJ; −1011, −19) but the differences should be viewed with caution given the small sample size (n = 15). The differences in the BMI z-score did not reach statistical significance (Table 3).

Table 4 demonstrates that ‘convenience’ and ‘packaging’ were the main reasons caregivers used baby food pouches across all age groups. ‘Health’ was the next most reported reason in infants, with ‘child enjoyment’ being the third most reported reason in toddlers and preschoolers. Interestingly, the ability to consume these products while ‘on-the-go’ was not provided as a common reason in any age group.

## 4. Discussion

Data from our large comprehensive observational study in a diverse sample of young children should allay some of the current concerns that expert groups have expressed about baby food pouches [7,8,9]. Only around one-quarter of infants aged 7 to 10 months were frequent consumers of baby food pouches, with most infants being fed the pouch contents from a spoon. Most infants were also fed using pouches while seated in a highchair or similar. Importantly, just 20 infants out of 503 who had consumed a baby food pouch (fewer than 4%) were frequent pouch consumers who regularly sucked the baby food directly from the pouch. Patterns were different in the older children, with nozzle sucking being the most common method in both toddlers and preschoolers, but the number of frequent consumers was low. Frequent consumption of baby food pouches was associated with lower total energy intakes in preschoolers, although ‘less frequent’ consumption was associated with higher energy intakes from complementary foods in infants. However, there was no significant relationship with BMI in any age group examined.

Data on market share support the popularity of baby food pouches worldwide, with 56% (USA) [3] and 64% (NZ) [1] of commercial infant foods being packaged in pouches. However, it is the frequency of consumption of baby food pouches that is more important because market share can only tell us about the relative popularity of pouches in relation to other commercial baby foods, and not about the prevalence and frequency of their use by young children in the community. We observed that just over a quarter of infants used baby food pouches frequently, with even lower numbers of toddlers and preschoolers being frequent consumers of these products. Very little data are available internationally for comparison. Finn et al. [10] reported that 11% of infants aged 6–11.9 months received food from a pouch at least daily in the US Feeding Infants and Toddlers study, broadly similar to our estimates of 19.2% for daily intake. Australian data indicated that 50% of children aged 0–24 months consumed food from a baby food pouch, but these data were obtained from a single day of 24-h recall assessment [30] rather than by a questionnaire assessing ‘usual’ consumption which may have contributed to the apparent higher prevalence. No other studies appear to have assessed baby food pouch intake in preschool-aged children, but it was apparent in the current study that these are still being consumed to some degree, even when children are 3 years of age. While fewer than 10% at this age could be classified as frequent users, consumption 1–4 times per week occurred in another 15.4% of preschoolers. This should perhaps not be surprising given the convenient nature of food pouches, and the fact that some are marketed, or at the very least appeal, to this age group.

Much of the concern about pouches appears based on the premise that infants will be regularly sucking the puréed foods through the pouch nozzle, with proposed impacts for nutritional health [7,8,9]. However, our novel findings indicate that while a number of infants do so at least occasionally, relatively few do so regularly, and regular consumption of baby food straight from the nozzle by frequent pouch users is rare, at fewer than 4%. This low frequency of exposure to “pouch sucking” by most infants does not appear in concordance with the level of concern expressed about pouches [8,9], at least in infants of this age. Although nozzle sucking appears more common in toddlers and preschoolers, as supported by the previously mentioned Australian data which also included toddlers [30], frequent pouch use overall is less common in our sample, such that the absolute number of toddlers and preschoolers who are both nozzle sucking and frequently using pouches is low.

Considerable concern has been expressed about the nutritional content of pouches, in particular the high level of sugars [1,8,31] that might predispose consumers to excess intake and overweight [7,32]. However, little work has examined whether this is indeed the case. Only one other study appears to have examined how baby food pouches that would be expected to be higher in sugars relate to weight status, showing that the consumption of fruit pouches was not related to BMI z-score at 18 months of age [11]. This is perhaps not surprising given that fruit pouches only represent a portion of the pouches available on the market. We have previously reported that the usual daily energy intake and BMI z-score did not differ in the FFNZ subsample of infants who were frequent consumers compared with those who did not consume them at all or did so less regularly [12]. We have also reported that although baby food pouches contributed a median of 47% of the total sugars infants consume in complementary foods, their overall contribution to energy intakes is relatively low. In infants, the median energy intake from baby food pouches was just 287 kJ a day or 25.5% of that obtained from complementary foods [33]. We now extend these findings to examine the energy intake from the total diet for toddlers and preschoolers, as well as for infants, and whether there are any potential dose–response effects with an increasing frequency of baby food pouch use. Interestingly, our data showed that infants who had consumed food from a pouch in the past month (either ‘less frequently’ or frequently), consumed about 10% more energy from complementary foods than non-consumers. However, this did not translate to significant differences in overall energy intake. This is likely due, at least in part, to the relatively small contribution baby food pouches make to the total energy intake in young children as presented here. Alternatively, our findings suggest some adjustment in infant milk intake, which perhaps explains the lack of any significant effect on BMI. It is also possible that because our study is cross-sectional, there may not have been sufficient time for any excess energy intake to become evident as a higher BMI. However, for many infants, using a pouch in the past month is likely to reflect longer term behaviour, given that 47% of infants were already using baby food pouches at 6 months of age. Similarly, although toddlers and preschool aged children who consumed pouches consumed less energy overall, and this was statistically significant for preschool frequent users, our numbers were small and no association with BMI was apparent.

Convenience and packaging were the most common reasons for using baby food pouches, which supports earlier consumer survey data from the US [6] and a recent ethnographic analysis [34]. Baby food pouches are often promoted for their ease and flexibility of use, which are features that would be expected to be compatible with the lifestyles of busy parents. Health and enjoyment were also commonly reported, whereas the portability of pouches, often considered one of the most appealing aspects of these products, was reported by few parents.

The strengths of this study include the novelty of the data, the measurement of health outcomes such as energy intake and body size, the large sample size, and the diversity of the population including large numbers of participants that were more socioeconomically disadvantaged, enabling good representation in the population estimates. Having robust data for these groups is important, given the inequities in health status among ethnic and socioeconomically deprived population groups [35,36]. However, several limitations must also be considered. First, some sampling bias may be present due to the use of convenience sampling, which may limit the generalizability of these findings. However, the sample population were ethnically diverse, with an even higher proportion of indigenous Māori people than is present in the wider population. By contrast, our proportion of Asian participants was lower than is observed nationally. Importantly, the levels of socioeconomic deprivation in this study reflected the national average in New Zealand very closely. Because of these minor differences we observed, we weighted the key results to population distributions for ethnicity and household deprivation to further reduce bias. Comparison of these results with the unweighted results shows little difference, highlighting the wide applicability of our findings. Because of the small numbers of infants in the YFNZ study, we did not weight the infant results for study (FFNZ or YFNZ), meaning that the estimates for infants are likely to be biased towards participants aged 7–10 months (n = 625 of the 645 total infant sample). Second, data were self-reported. Consequently, social desirability bias in reporting may be present (e.g., adult participants may have over-reported how often the infant was supervised by an adult when using a pouch). Third, this study was carried out in a population of infants and young children from New Zealand, so findings may not be generalizable to other countries due to different food supplies and contexts. Fourth, in the absence of any established definitions, we defined frequent pouch use as baby food pouch use 5+ times per week, but this does not consider the amount of food eaten from the pouch.

## 5. Conclusions

In conclusion, the use of baby food pouches, on at least an occasional basis, is common in New Zealand infants. However, the low prevalence of regular consumption directly through the nozzle by frequent users does not appear to support concerns about their use. We saw no adverse effects on energy intake and body size in these cross-sectional data, but further research is needed regarding other health outcomes of interest.

## Figures and Tables

**Table 1:** Child demographic characteristics and growth measures.

	Infants ^a^	Toddlers ^a^	Young Preschool Children ^a^	Total Sample
n	645	106	182	933
Age, mean (SD) months	8.4 (0.9)	18.3 (3.6)	36.8 (6.8)	15.1 (11.6)
Sex, n (%)				
Female	297 (46.1)	57 (53.8)	91 (50.0)	445 (47.7)
Male	347 (53.8)	49 (46.2)	91 (50.0)	487 (52.2)
Not specified	1 (0.2)	0	0	1 (0.1)
Area-level socioeconomic deprivation ^b^, n (%)				
1 to 3 (Low)	188 (29.2)	25 (23.6)	55 (30.2)	268 (28.7)
4 to 7	288 (44.7)	46 (43.4)	78 (42.9)	412 (44.2)
8 to 10 (High)	169 (26.2)	35 (33.0)	49 (26.9)	253 (27.1)
Ethnicity ^c^, n (%)				
Māori	136 (21.1)	32 (30.2)	42 (23.1)	210 (22.5)
Pacific	49 (7.6)	18 (17.0)	29 (15.9)	96 (10.3)
Asian	91 (14.1)	11 (10.4)	26 (14.3)	128 (13.7)
Others	17 (2.6)	1 (0.9)	4 (2.2)	22 (2.4)
European	352 (54.6)	44 (41.5)	81 (44.5)	477 (51.1)
Currently breastfed	426 (66.1)	43 (40.6)	12 (6.6)	481 (51.6)
Length/height ^d^, mean (SD) cm	70.8 (3.2)	82.1 (5.8)	96.9 (5.9)	76.7 (11.0)
Weight ^d^, mean (SD) kg	8.8 (1.1)	11.5 (2.0)	15.6 (2.4)	10.3 (3.1)
BMI ^d^, mean (SD) kg/m^2^	17.5 (1.5)	17.1 (1.8)	16.5 (1.4)	17.3 (1.6)
BMI z-score ^d^	0.29 (0.99)	0.72 (1.21)	0.72 (1.02)	0.41 (1.04)

^a^ Infants: 6.0 to 11.9 months; Toddlers: 12.0 to 23.9 months; Preschool children: 24.0 to 47.9 months. ^b^ Using the New Zealand Index of Deprivation (NZDep18) (index based on census data that reflect the extent of material and social deprivation used to construct deciles from 1 (least deprived) to 10 (most deprived) [16]). ^c^ Using prioritised ethnicity from Census Level 2 reporting [15] with prioritisation in the order displayed in the table. ^d^ 51 participants missing length/height (11 older infants, 21 toddlers, and 19 preschool children); 53 participants missing weight (13 older infants, 21 toddlers, and 19 preschool children); 59 participants missing BMI (19 older infants, 21 toddlers, and 19 preschool children); 60 participants missing BMI z-score (20 older infants, 21 toddlers, and 19 preschool children). BMI z-scores determined using the WHO Child Growth Standards [27].

**Table 2:** Pouch use according to age.

	Infants ^a^	Toddlers ^a^	Young Preschool Children ^a^	Total Sample
n	645	106	182	933
Ever had a baby food pouch	506 (78.5)	93 (87.7)	153 (84.1)	752 (80.6)
Frequency of consumption in the past month				
Never	162 (25.1)	36 (34.0)	114 (62.6)	312 (33.4)
3 or fewer times/month	145 (22.5)	28 (26.4)	25 (13.7)	198 (21.2)
1–4 times/week	161 (25.0)	25 (23.6)	28 (15.4)	214 (22.9)
5–6 times/week	53 (8.2)	4 (3.8)	3 (1.7)	60 (6.4)
1 or more times/day	124 (19.2)	13 (12.3)	12 (6.6)	149 (16.0)
Frequent pouch use categories ^b^				
Non-users	162 (25.1)	36 (34.0)	114 (62.6)	312 (33.4)
Less frequent users	306 (47.4)	53 (50.0)	53 (29.1)	412 (44.2)
Frequent users	177 (27.4)	17 (16.0)	15 (8.2)	209 (22.4)
How pouches were fed				
n	503	93	148	744
Always by the nozzle	50 (9.9)	56 (60.2)	101 (68.2)	207 (27.8)
Mostly by the nozzle	37 (7.4)	15 (16.1)	17 (11.5)	69 (9.3)
About half nozzle, half spoon	36 (7.2)	7 (7.5)	10 (6.8)	53 (7.1)
Mostly by spoon	91 (18.1)	9 (9.7)	5 (3.4)	105 (14.1)
Always by spoon	289 (57.5)	6 (6.5)	15 (10.1)	310 (41.7)
How pouches are fed in frequent pouch users				
n	176	17	15	208
Always by the nozzle	20 (11.4)	9 (52.9)	14 (93.3)	43 (20.7)
Mostly by the nozzle	11 (6.3)	5 (29.4)	0	16 (7.7)
About half nozzle, half spoon	15 (8.5)	2 (11.8)	0	17 (8.2)
Mostly by spoon	34 (19.3)	1 (5.9)	0	35 (16.8)
Always by spoon	96 (54.6)	0	1 (6.7)	97 (46.6)
Usual location of pouch consumption in the last month				
n	474	70	67	611
Sitting in a seat ^c^	355 (74.9)	37 (52.9)	33 (49.3)	425 (69.6)
While on-the-go ^d^	56 (11.8)	25 (35.7)	25 (37.3)	106 (17.4)
On someone’s knee	34 (7.2)	1 (1.4)	2 (3.0)	37 (6.1)
Sitting on the floor	19 (4.0)	7 (10.0)	3 (4.5)	29 (4.8)
At childcare	10 (2.1)	0	4 (6.0)	14 (2.3)
Frequency of supervision while pouch consumed in the last month				
n	480	70	67	617
Never	4 (0.8)	3 (4.3)	10 (14.9)	17 (2.8)
Sometimes	13 (2.7)	9 (12.9)	23 (34.3)	45 (7.3)
About half	7 (1.5)	6 (8.6)	11 (16.4)	24 (3.9)
Almost always	38 (7.9)	28 (40.0)	13 (19.4)	79 (12.8)
Always	418 (87.1)	24 (34.3)	10 (14.9)	452 (73.3)

^a^ Infants: 6.0 to 11.9 months; Toddlers: 12.0 to 23.9 months; Preschool children: 24.0 to 47.9 months. ^b^ Frequent pouch use categories defined as: ‘Non-users’, no use in the last month; ‘Less frequent users’, used less than 5 times a week in the last month; ‘Frequent users’, used at least 5 times a week in the last month. ^c^ Includes ‘highchair’ and ‘chair’. ^d^ Includes in the ‘car’, in a ‘buggy or pram’, and while ‘on-the-go’.

**Table 3:** Energy intake and BMI z-score according to frequency of pouch use.

	Non-Users ^a^	Less Frequent ^a^	Frequent ^a^
Infants ^b^			
Usual daily energy intake, kJ			
n	162	305	177
Mean (SD)	3218 (585)	3192 (699)	3333 (795)
Mean difference (95% CI)	Reference	−26 (−160, 108)	115 (−35, 265)
Adjusted mean difference ^c^ (95% CI)	Reference	−38 (−170, 94)	106 (−42, 255)
Energy intake from complementary foods, kJ			
n	162	306	177
Mean (SD)	1164 (765)	1336 (728)	1318 (813)
Mean difference (95% CI)	Reference	173 (28, 318)	154 (−8, 317)
Adjusted mean difference ^c^ (95% CI)	Reference	137 (10, 264)	120 (−23, 263)
BMI z-score			
n	157	299	169
Mean (SD)	0.20 (1.05)	0.28 (0.95)	0.38 (1.02)
Mean difference (95% CI)	Reference	0.08 (−0.11, 0.27)	0.18 (−0.04, 0.40)
Adjusted mean difference ^c^ (95% CI)	Reference	0.07 (−0.12, 0.27)	0.17 (−0.05, 0.39)
Toddlers ^b^			
Usual daily energy intake, kJ			
n	36	53	17
Mean (SD)	4576 (888)	4165 (976)	4370 (1270)
Mean difference (95% CI)	Reference	−411 (−839, 17)	−206 (−789, 378)
Adjusted mean difference ^c^ (95% CI)	Reference	−325 (−753, 101)	−42 (−635, 551)
BMI z-score			
n	26	44	15
Mean (SD)	0.95 (1.10)	0.59 (1.19)	0.68 (1.45)
Mean difference (95% CI)	Reference	−0.37 (−0.96, 0.23)	−0.28 (−1.06, 0.51)
Adjusted mean difference ^c^ (95% CI)	Reference	−0.37 (−0.99, 0.25)	−0.16 (−1.00, 0.68)
Preschoolers ^b^			
Usual daily energy intake, kJ			
n	114	52	15
Mean (SD)	5315 (951)	5149 (863)	4735 (1195)
Mean difference (95% CI)	Reference	−166 (−479, 147)	−580 (−1094, −67)
Adjusted mean difference ^c^ (95% CI)	Reference	−80 (−384, 224)	−515 (−1011, −19)
BMI z-score			
n	103	47	13
Mean (SD)	0.81 (0.97)	0.55 (1.04)	0.57 (1.24)
Mean difference (95% CI)	Reference	−0.25 (−0.61, 0.10)	−0.24 (−0.83, 0.35)
Adjusted mean difference ^c^ (95% CI)	Reference	−0.26 (−0362, 0.11)	−0.27 (−0.87, 0.32)

^a^ Frequent pouch use categories defined as: ‘Non-users’, no use in the last month; ‘Less frequent users’, used less than 5 times a week in the last month; ‘Frequent users’, used at least 5 times a week in the last month. ^b^ Infants: 6.0 to 11.9 months; Toddlers: 12.0 to 23.9 months; Preschool children: 24.0 to 47.9 months. One participant did not have diet recall data in the toddlers group. ^c^ Associations adjusted for age and area-level socioeconomic deprivation.

**Table 4:** Reasons respondents used a food pouch.

	Infants ^a^	Toddlers ^a^	Young Preschool Children ^a^	Total Sample
n	483	70	68	621
Convenience ^b^	372 (77.0)	45 (64.3)	41 (60.3)	458 (73.8)
Packaging ^c^	326 (67.5)	50 (71.4)	44 (64.7)	420 (67.6)
Child enjoys ^d^	180 (37.3)	40 (57.1)	43 (63.2)	263 (42.4)
Health ^e^	207 (42.9)	19 (27.1)	20 (29.4)	246 (39.6)
On the go ^f^	53 (11.0)	6 (8.6)	1 (1.5)	60 (9.7)
Others ^g^	10 (2.1)	1 (1.4)	0	11 (1.8)

^a^ Infants: 6.0 to 11.9 months; Toddlers: 12.0 to 23.9 months; Preschool children: 24.0 to 47.9 months. ^b^ Convenience reasons included the following: ‘practical’, no time to cook, no homemade available, no food prepared, emergency meal, disorganised, hands-free, takes less time, cooking not needed, availability, and laziness. ^c^ Packaging reasons included the following: environmentally friendly, packaging keeps it fresh, safety, easy to use, resealable, easy storage, chilling not needed, and less mess. ^d^ Child enjoys reasons included the following: treat, snack, adds taste, baby dislikes alternative, only food baby eats, and child likes. ^e^ Health reasons included the following: gives variety/diversity of foods, organic, good food for baby, healthier than family meals, easy to get meat in, easy to get fruits and/or vegetables in, easy to get food in, low sugar custard, fibre, allergen foods, mix with medicine, heard they were good, and constipation. ^f^ On the go reasons included the following: for travel, when dining out, easy for transport, and convenient for childcare. ^g^ Other reasons included the following: ‘freebie’ (n = 6), used for baby-led weaning (n = 2), giving it a try (n = 5), and ‘no reason’ (n = 3).

## Data Availability

De-identified data can be obtained from the corresponding author upon reasonable request.
